# Subcutaneous Infection with *Dirofilaria immitis* Nematode in Human, France

**DOI:** 10.3201/eid1901.120281

**Published:** 2013-01

**Authors:** Maud Foissac, Matthieu Million, Charles Mary, Jean-Philippe Dales, Jean-Baptiste Souraud, Renaud Piarroux, Philippe Parola

**Affiliations:** Author affiliations: Assistance Publique–Hôpitaux de Marseille, Marseille, France (M. Foissac, M. Million, C. Mary, J.-P. Dales, R. Piarroux, P. Parola);; Aix-Marseille Université, Marseille (M. Foissac, M. Million, C. Mary, J.-P. Dales, P. Parola);; Hôpital d’Instruction des Armées, Sainte-Anne, Toulon, France (J.-B. Souraud)

**Keywords:** Dirofilaria repens, Dirofilaria immitis, parasites, nematode, subcutaneous nodule, subcutaneous infection, human, PCR, France

**To the Editor:** In March 2012, a 48-year-old woman was hospitalized with a subcutaneous nodule on her right thigh that was present for 4 weeks. She was living in Martigues near Marseille in southeastern France, owned cats and dogs, and never traveled out of France. Ultrasonography examination showed a diffuse subcutaneous edema without abscess. Results of initial blood count; ionogram; and tests for urea, creatinine, and liver enzyme levels were within reference ranges, but slight hypereosinophilia (0.7 × 10^9^ cells/L) was noted 1 month later. Serologic results for toxocariasis, schistosomiasis, trichinosis, distomatosis, cysticercosis, and microfilaremia (3 assays) were negative. However, the result of an ELISA for *Dipetalonema viteae* nematodes was positive.

After surgical removal of the nodule, histopathologic analysis showed a female nematode surrounded by an aspecific inflammatory reaction. Transverse sections showed a parasite with a diameter of 300 µm and a layered cuticle <20 µm thick. The surface of the cuticle had numerous external, cuticular, longitudinal ridges. The nematode had 2 uterine tubules without microfilaremia, 1 intestinal tube, and a polymyarian-type musculature interrupted by 2 lateral chords. This worm showed similarities to an immature *Dirofilaria repens* female.

To identify the worm, we performed PCR amplification of a 12S rRNA gene fragment (*1*). PCR products (International Nucleotide Sequence Database Collaboration accession no. JX502021) were compared with sequences deposited in GenBank. Analysis showed 92% similarity and 89% coverage with the 12S rRNA gene of the *D. repens* nematode reference sequence (GenBank accession no. GQ292761.1) (*2*) but 100% similarity and 100% coverage with the 12S rRNA gene of the *Dirofilaria immitis* nematode reference sequence (GenBank accession no. EU169125.1) (*3*).

To confirm this result, we amplified cytochrome c oxidase 1 (*cox-1*) and internal transcribed spacer 2 (ITS-2) genes by using a duplex real-time PCR as described (*4*). Results were positive for *D. immitis* (International Nucleotide Sequence Database Collaboration accession no. HE979797 [100% similarity and 100% coverage with the *cox-1* gene of the *D. immitis* reference sequence] and GenBank accession no. JF461464.1) (*5*) and negative for the *D. repens* ITS-2 gene. These results confirmed an unexpected *D. immitis* subcutaneous infection that would have been misdiagnosed without molecular analysis.

*D. repens* and *D. immitis* nematodes are the most common species causing dirofilariasis in temperate and tropical areas. Dogs and cats are usual hosts, and main vectors are *Aedes*, *Culex*, and *Anopheles* spp. mosquitoes (*6*). In dogs and cats, *D. immitis* nematodes cause severe infections that affect lung vessels and heart cavities. In humans, this nematode is mainly responsible for benign asymptomatic pulmonary nodules, but *D. repens* nematodes usually induce periocular or subcutaneous lesions, as in our patient (*6*).

Until 2001, three areas in Europe (Iberian Peninsula, southern France, and Italy) were greatly affected by dirofilariasis. Its incidence has since increased in animals, and epidemiologic surveys showed spread of both nematode species to areas previously free of *Dirofilaria* nematodes, such as Germany and eastern Europe (Romania, Croatia, Serbia, Bulgaria, Czech Republic, and Rostov region in Russia) (*7*). Increased transport of microfilaremic pets throughout Europe, building construction and other human activities in new areas, emergence of new competent vectors (such as highly adaptable *Ae. albopictus* mosquitoes), and climate changes affected this spread (*6*). Climate changes indirectly influenced abundance of mosquitoes in specific areas, their period of activity, and development of *Dirofilaria* nematodes in vectors (*6*). Thus, incidence of human dirofilariasis is also expected to increase, although many asymptomatic infections are not diagnosed.

Fewer than 30 cases of human *D. immitis* infections have been reported in Europe since 1981, including only 4 in France. Moreover, 16 of these cases were questioned by Pampiglione et al. (*8*) because of unreliable diagnostic tools (serologic testing without a negative control) or atypical histologic criteria. Although other cases attributed to *D. immitis* nematodes have been reported, we found only 1 case in Europe attributed to *D. immitis* nematodes for which the diagnosis was confirmed by PCR (*9*).

Definitive diagnosis of dirofilariasis remains a difficult challenge. Noninvasive tests (mainly dirofilarial serologic assays) lack sensitivity, specificity, and standards to be considered reliable methods (*6*). Until recently, the standard test was histologic identification on the basis of diameter of the nematode, thickness of the cuticle, number and distribution of the fibers in the muscular layer, and study of external cuticular ridges (*8*). However, diagnosis by histopathologic analysis may be unreliable if worms are immature or subjected to necropsy, and *D. immitis* nematodes may be misidentified as *D. repens* nematodes.

Our case indicates the difficulties with histopathologic analysis (e.g., the nematode showed similarity with *D. repens* nematodes because of external cuticular ridges). However, using validated molecular techniques (*4*,*5*), we showed that infection with *D. immitis* nematodes would have been erroneously identified as infection with *D. repens* nematodes. Thus, we believe that PCR-based identification should be considered as a new diagnostic method for dirofilariasis.
